# Ultra-sensitive CTC-based liquid biopsy for pancreatic cancer enabled by large blood volume analysis

**DOI:** 10.1186/s12943-023-01880-1

**Published:** 2023-11-13

**Authors:** Nikolas H. Stoecklein, Georg Fluegen, Rosa Guglielmi, Rui P.L. Neves, Thilo Hackert, Emrullah Birgin, Stefan A. Cieslik, Monica Sudarsanam, Christiane Driemel, Guus van Dalum, André Franken, Dieter Niederacher, Hans Neubauer, Tanja Fehm, Jutta M. Rox, Petra Böhme, Lena Häberle, Wolfgang Göring, Irene Esposito, Stefan A. Topp, Frank A.W. Coumans, Jürgen Weitz, Wolfram T. Knoefel, Johannes C. Fischer, Ulrich Bork, Nuh N. Rahbari

**Affiliations:** 1 General, Visceral and Pediatric Surgery, University Hospital and Medical Faculty of the Heinrich-Heine-University Düsseldorf, Moorenstr. 5, 40225 Düsseldorf, Germany; 2 Department of General, Visceral, and Transplantation Surgery, Heidelberg University Hospital, Im Neuenheimer Feld 420, 69120 Heidelberg, Germany; 3 Department of General, Visceral and Thoracic Surgery, University Medical Center Hamburg-Eppendorf, Martinistr. 52, 20246 Hamburg, Germany; 4 Department of Surgery, Medical Faculty Mannheim, University Hospital Mannheim, Theodor-Kutzer-Ufer 1-3, 68167 Mannheim, Germany; 5 Department of Obstetrics and Gynecology, University Hospital and Medical Faculty of the Heinrich-Heine-University Düsseldorf, Moorenstr. 5, 40225 Düsseldorf, Germany; 6 Department of Transplantation Diagnostics and Cell Therapeutics, University Hospital and Medical Faculty of the Heinrich-Heine-University Düsseldorf, Moorenstr. 5, 40225 Düsseldorf, Germany; 7 Institute of Forensic Medicine Düsseldorf, University Hospital and Medical Faculty of the Heinrich-Heine-University Düsseldorf, Moorenstr. 5, 40225 Düsseldorf, Germany; 8 Institute of Pathology, University Hospital and Medical Faculty of the Heinrich-Heine-University Düsseldorf, Moorenstr. 5, 40225 Düsseldorf, Germany; 9 Decisive Science, Ertskade 10, 1019 BB, Amsterdam, The Netherlands; 10 Current Affiliation: Department for General and Visceral Surgery, University Hospital Ulm, Albert-Einstein-Allee 23, 89081 Ulm, Germany; 11 Department of Visceral, Thoracic and Vascular Surgery, University Hospital Carl Gustav Carus of the Technical University Dresden, Fetscherstr. 74, 01307 Dresden, Germany

**Keywords:** Pancreatic ductal adenocarcinoma, PDAC, Circulating tumor cells, CTCs, High-blood volume analysis, Liquid biopsy, Diagnostic leukapheresis, DLA, Single cell genomics, CTC profiling

## Abstract

The limited sensitivity of circulating tumor cell (CTC) detection in pancreatic adenocarcinoma (PDAC) stems from their extremely low concentration in the whole circulating blood, necessitating enhanced detection methodologies. This study sought to amplify assay-sensitivity by employing diagnostic leukapheresis (DLA) to screen large blood volumes. Sixty patients were subjected to DLA, with a median processed blood volume of ~ 2.8 L and approximately 5% of the resulting DLA-product analyzed using CellSearch (CS). Notably, DLA significantly increased CS-CTC detection to 44% in M0-patients and 74% in M1-patients, yielding a 60-fold increase in CS-CTC enumeration. DLA also provided sufficient CS-CTCs for genomic profiling, thereby delivering additional genomic information compared to tissue biopsy samples. DLA CS-CTCs exhibited a pronounced negative prognostic impact on overall survival (OS), evidenced by a reduction in OS from 28.6 to 8.5 months (univariate: p = 0.002; multivariable: p = 0.043). Additionally, a marked enhancement in sensitivity was achieved (by around 3-4-times) compared to peripheral blood (PB) samples, with positive predictive values for OS being preserved at around 90%. Prognostic relevance of CS-CTCs in PDAC was further validated in PB-samples from 228 PDAC patients, consolidating the established association between CTC-presence and reduced OS (8.5 vs. 19.0 months, p < 0.001). In conclusion, DLA-derived CS-CTCs may serve as a viable tool for identifying high-risk PDAC-patients and aiding the optimization of multimodal treatment strategies. Moreover, DLA enables comprehensive diagnostic profiling by providing ample CTC material, reinforcing its utility as a reliable liquid-biopsy approach. This high-volume liquid-biopsy strategy presents a potential pathway for enhancing clinical management in this malignancy.

**Supplementary Information:**

The online version contains supplementary material available at 10.1186/s12943-023-01880-1.

## Introduction

Pancreatic ductal adenocarcinoma (PDAC) is characterized by a high mortality-to-incidence ratio and is projected to become the second leading cause of cancer-related death in Western countries by 2030 [1]. Only 15–20% of newly diagnosed patients have localized disease and are eligible for curative surgical treatment [2]. Despite surgery with curative intent, patients can only expect a 5-year overall survival rate of around 20–25% due to occurrence of metastatic disease, the most common kind of recurrence [3–5]. Therefore, biomarkers are urgently needed to identify patients with clinically occult systemic PDAC at the timepoint of surgery in order to select high-risk patients for more intense regimens or to avoid major surgery in patients unlikely to benefit.

In this context, circulating tumor cells (CTCs) are promising biomarkers for PDAC, as they can represent active systemic cancer [6]. The current gold standard for CTC detection is the FDA cleared CellSearch (CS) system, which defines CTCs as EpCAM+/CK+/CD45−/DAPI + cells (CS-CTC). While CS-CTC detection is high in advanced prostate or breast cancer, it is surprisingly low in PDAC, with approximately 10% in M0 patients and 40% in M1 patients [7, 8]. Compared to prostate and breast cancer, only few studies with relatively small cohorts have been reported on CS in PDAC. The data suggested that CS-CTCs may identify operable PDAC patients at high risk for poor survival [7, 8]. However, the low detection frequencies clearly indicate that a significant number of high-risk patients escape detection, limiting the utility for patient selection or CTC-based diagnostic profiling. To address these issues, we employed an ultra-sensitive, high-blood-volume approach to enhance CS-CTC detection via leukapheresis.

Our objective was to demonstrate the feasibility of identifying high-risk patients in early PDAC stages and to enable the harvest of a sufficient quantity of CTCs for liquid biopsy-based profiling, thereby complementing tissue biopsies in cases of inoperable disease. According to our hypothesis, the low CTC detection is mainly due to an insufficient sample volume relative to the total body volume. Statistical models accounting for the Poisson distribution of rare CTCs calculated that the analysis of 1–5 L of blood should enable the detection of 10–50 CTCs in virtually every metastatic cancer patient [9, 10]. As a first step to address this challenge, we introduced diagnostic leukapheresis (DLA) [11], a shortened leukapheresis protocol to harvest a white blood cell (WBC) fraction (i.e. MNCs) based on their specific density from more than two liters of blood by continuous centrifugation. Since density profiles of epithelial cancer cells overlap with the targeted MNCs [12], CTCs become co-collected along the process. Previous studies have demonstrated the feasibility of using DLA for CTC enrichment in patients with advanced breast, prostate, and lung cancers [13–16]. Therefore, we sought to investigate whether DLA could improve the sensitivity of CS-CTC detection and consequently enhance identification of high-risk patients PDAC.

## Results and discussion

### DLA significantly improves detection of CS-CTCs in PDAC

**Fig. 1:**
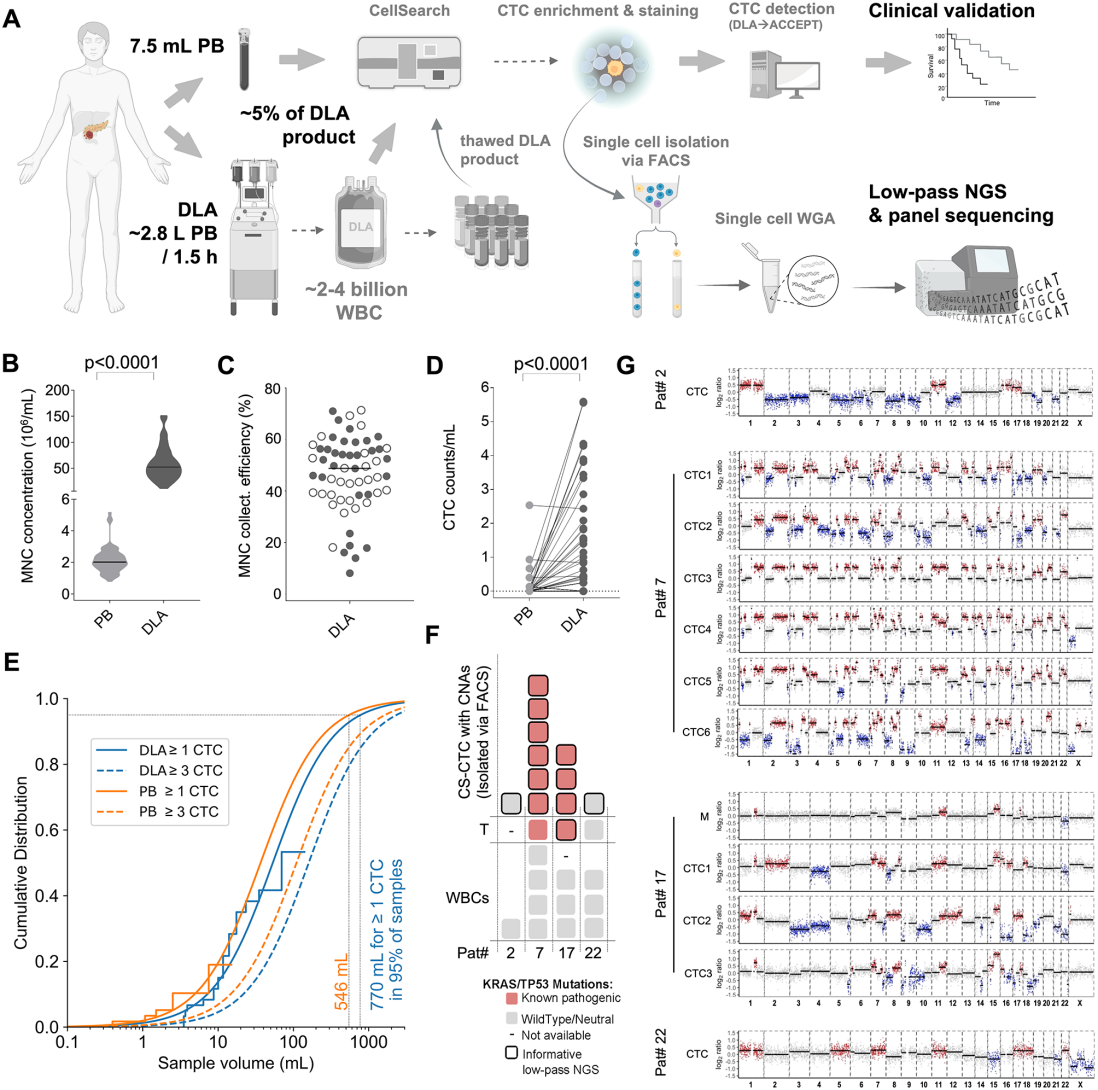
Study overview and DLA findings: **A** Experimental setup (created in part with BioRender.com); **B** MNCs per mL in peripheral blood samples (D-PB, n = 60) versus matched DLA samples (D-DLA, n = 60); **C** Collection efficacy achieved for the targeted MNC population (n = 60). The filled circles indicate CTC-positivity (≥ 1 CTC detected); **D** CTC-concentration per mL in PB samples (light grey circles) and matched DLA samples (dark grey circles); **E**. Extrapolation of log-logistic fit curves for peripheral blood samples (PB) and DLA samples (DLA). Stair plots show the empirical CDF and the continuous line the fit. Extrapolation indicates probability (Cumulative Distribution) for a certain analyzed sample volume to have at least 1 CTC or 3 CTCs in every sample; **F** Genomic analysis of individual CS-CTCs isolated from DLA products with panel sequencing of KRAS and TP53 genes (T: tumor tissue); **G** Plots of copy number profiles along the 22 autosomes expressed as log2 ratios. Significant copy number gains and losses are highlighted in red and blue, respectively (M: FFPE material from metastatic tissue)

Please refer to Additional File 1, Supplementary Methods for the materials and methods of this study. To explore the effects of DLA for PDAC CTCs, we followed the workflow illustrated in Fig. 1A. DLA with sufficient MNC collection efficacy (median: 49%; range: 8–71%; Fig. 1B-C) for this purpose was performed in 60 PDAC patients (DU-DLA-cohort) without technical issues or notable adverse effects. Although lower efficacies also produced CS-CTC positive samples, DLAs with a collection efficacy equal to or above the median resulted in a higher CS-CTC-detection frequency (11/30 vs. 21/30; Fisher’s exact test: p = 0.02, Fig. 1C). From the processed median blood volume of ~ 2.8 L (2,795mL, range: 769-5,765mL) we generated DLA products with a median volume of 45mL (range: 10-100mL). The capacity of the CS-assay required limiting the sample input to around 200*10^6^ WBC [11], which represented approximately 5% of a DLA product. The remaining material was cryopreserved as aliquots. The calculated CS-input corresponded to a median blood volume of ~ 70mL (70.6mL, range: 20.50mL-181.03mL) (Additional File 1, Supplementary Fig. 1). Applying this process, we observed a significantly increased detection frequency of 53% (32/60) in DLA compared to 19% in matched PB samples (11/58; Fisher’s Exact Test, p < 0.001). With 1.05 (±1.51) CTC/mL the mean CS-CTC concentration was 10-times higher in DLA compared to 0.10 (±0.37) CTC/mL in PB (Fig. 1D). To estimate the overall CTC-load in DLA products, we extrapolated the detected CS-CTCs to the entire DLA product, revealing a calculated median of 71.5 CTCs (range: 0-218) per DLA product and a 60-fold (mean) increase in CTC numbers compared to PB. This enrichment suggests that PDAC-CTCs fall into the density window targeted by our DLA settings. Next, we aimed to determine the DLA-product volume, required to analyze for detecting at least one CS-CTC in every PDAC patient. Fitting our PB and DLA data to a distribution function described previously for PB [9], we ascertained that the distribution function also holds for the larger volumes of the DLA product. Furthermore, we extrapolate that analyzing a DLA product with a corresponding median blood volume of 1000mL is expected to yield 1 CTC in 96% of samples, 3 CTC in 88% of samples and 10 CTC in 67% of samples (Fig. 1E). This volume is equivalent to approximately 76% of the DLA product (34mL). The 30% discrepancy between the volumes derived from PB samples (7.5mL) and DLA samples (~ 70mL) indicates the CTC loss incurred in the DLA sample preparation process as compared to PB. However, it is important to stress that forthcoming technological advancements [10, 17], proficient in managing the designated DLA volumes, should feasibly enable processing of the required DLA-product volume in an imminent time frame.

### Uncovering the genomic alterations of PDAC CTCs through DLA-enabled profiling

Owing to their rarity in PB, genomic data on CS-CTCs in PDAC have remained unavailable until now. To demonstrate the feasibility of employing DLA-enriched CS-CTCs as a surrogate for molecular profiling and to validate their malignant character, we re-analyzed cryopreserved material from CS-CTC-positive DLA samples. Notably, the quantity of CS-CTCs in the frozen DLA samples was not significantly deviant from those freshly and prospectively analyzed (Additional File 1, Supplementary Fig. 2). These results affirm that processing the entire DLA product could further elevate the sensitivity of our approach, as was suggested by our extrapolation. After isolation, we conducted genomic profiling of 11 single CS-CTCs and matched tumor tissue of four patients (#2, #7, #17, #22), including one patient (#22) in whom relevant copy number alteration (CNAs) were not detected in the tumor tissue (Fig. 1F-G; Additional File 1, Supplementary Table 4). In two patients (#2 and #22), KRAS and TP53 mutations were not detected in either the CTCs harboring CNAs or the matched tumor tissue. Patient #7 exhibited consistent detection of a G12V mutation in both CTCs and tumor tissue that displayed an additional G12D mutation (Fig. 1F-G; Additional File 1, Supplementary Table 4). In patient #17, CTCs showed a G12V mutation while a G12R mutation was observed in the matched tumor tissue (Additional File 1, Supplementary Table 4). As a control, we analyzed 26 matched and non-matched white blood cells isolated from CS cartridges of thawed PDAC DLA samples and did not detect any pathogenic mutations (Additional File 1, Supplementary Table 4). The different hot-spot mutations between tumor tissue and CTCs prompted us to perform subsequent short tandem-repeat analysis (STR), which authenticated the sample identity (data not shown). Collectively, this exemplary genomic CTC-profiling not only confirmed the malignant nature of DLA-enriched CS-CTCs but also demonstrated the feasibility of performing comprehensive genomic analysis on CS-CTCs from DLA-products, providing additional information compared to PDAC tissue. Considering the recognized intra-patient molecular heterogeneity, employing their versatile analysis could offer substantial advantages over classical tissue biopsies in metastatic PDAC. This is particularly pertinent in the context of emerging molecular therapies, which will necessitate diagnostic molecular tumor profiling [2, 18, 19].

### DLA enriched CS-CTCs are linked to higher tumor stage and poor survival

**Fig. 2:**
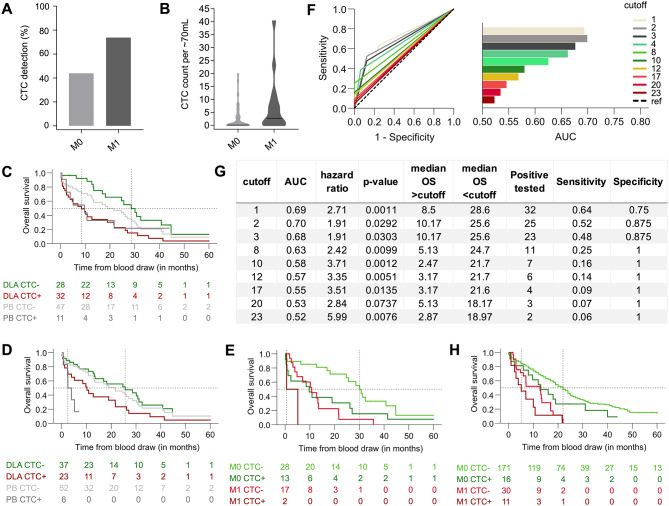
Association of PDAC CS-CTC with clinical parameters. **A** Frequency of CTC-positivity (≥ 1 CTC detected) in DLA products according to the metastatic stage; **B** Normalized CS-CTC-concentration in DLA samples according to the metastatic stage; **C-D** OS according to status of DLA CS-CTC (n = 60, CTC/~70mL) in green/red compared to PB CS-CTC (n = 58, CTC/7.5mL blood) in grey. **C** Cutoff for CTC positivity: ≥1 CTC; **D** Cutoff for CTC positivity: ≥3 CTC; **E** OS according to the CTC-status separated by metastatic stage in DLA products (optimal cutoffs: M0 = 3CTC/~70mL blood; M1 = 23CTC/~70mL blood, see Additional File 1, Supplementary Fig. 4); **F** ROC curves and corresponding AUC values for the top 10 DLA CS-CTC cutoffs normalized to ~ 70mL of blood. These cutoffs were selected based on their combined highest hazard ratio and AUC value in the DLA cohort of 60 PDAC patients (ref: reference); **G** Parameters for all significant (p < 0.05) DLA CS-CTCs cutoff values calculated in the cohort of 60 PDAC patients (Cutoff: CTCs/~70mL); **H** OS according to CS-CTC status (≥ 1CTC/7.5mL blood) in PB pooled HE/DU-PB cohorts separated by metastatic stage (M0 vs. M1)

To validate the clinical relevance of CS-CTCs enriched by DLA, we examined their association with clinical parameters. Given the individual variations in assay input (Additional File 1, Supplementary Fig. 1), CTC counts were normalized to the median analyzed blood-equivalent of ~ 70mL, with resulting decimal numbers rounded to the nearest integer (range: 0–40 CTCs). Although CTC count normalization was crucial for accurate concentration and survival analyses, notable differences in detection frequencies between M0 and M1 patients were evident without this adjustment. For DLA samples, detection rates were 18/41 for M0 patients and 14/19 for M1 patients (Fisher’s exact test: p = 0.0507, see Fig. 2A). Meanwhile, for PB samples, the detection rate was 4/41 for M0 patients and 7/17 for M1 patients (Fisher’s exact test: p = 0.0099). Notably, only DLA samples demonstrated a significantly higher CS-CTC concentration in M1 patients compared to M0 patients (median: 3.0 vs. 0.0, Mann-Whitney U test: p = 0.024, Fig. 2B). Subsequently, we tested the prognostic value of DLA-enriched CS-CTCs and adopted a cut-off of ≥ 1CTC per ~ 70mL, aligning with thresholds used in existing PDAC studies involving CS [7, 8]. Additionally, to determine a more robust threshold for DLA, we incorporated ≥ 3CTC/~70mL, considering a ~ 5% probability of detecting 0 CTC with an average detection frequency/threshold of 3 CTCs per sample versus a ~ 36% probability at a mean detection frequency/threshold of 1 CTC per sample, due to the Poisson distribution. Strikingly, DLA-derived CS-CTC revealed prognostic significance at both thresholds, with one CTC per sample reducing median overall survival (OS) to 8.5 months, compared to 28.6 months without CS-CTC (log-rank test: p = 0.002; Fig. 2C), and ≥ 3CTCs reducing OS to 10.2 months vs. 25.6 months below this threshold (log-rank test: p = 0.033; Fig. 2D). Upon multivariable analysis, the adverse effect of the threshold of ≥ 1CTC retained its significance, underscoring prognostic relevance. The prognostic effects of DLA CS-CTC outperformed those observed for CS-CTC in matched PB samples (Fig. 2C **and D**, Additional File 1, Supplementary Table 5), especially concerning sensitivity and accuracy in identifying patients at high risk for short OS (Additional File 1, Supplementary Table 8). The superior prognostic impact of DLA CS-CTCs was also evident in M0 patients and those treated with curative intent (Additional File 1, Supplementary Fig. 3, and Supplementary Tables 6–8). Notably, a relatively high OS-rate was apparent for M0 patients without DLA CS-CTC (e.g., at 20 months), while DLA CS-CTC-positive M0 patients exhibited similarly poor OS compared with CS-CTC-negative M1 patients (Fig. 2E). As anticipated, CS-CTC-positive M1-patients displayed the worst prognosis. Interestingly, an analysis for CTC-cutoff values, which considered both hazard ratios and AUC values collectively (Fig. 2F **and G**), confirmed our tested cutoffs as optimal. Expectedly, elevating CTC-cutoff values enhanced specificity while diminishing sensitivity. Further validation of the prognostic value of CS-CTCs in PDAC was sought through analyzing PB samples in an independent, large cohort (HE: n = 170) and in combination with the DU-PB-cohort (n = 228; Fig. 2H, Additional File 1, Supplementary Table 9), affirming the significant and independent prognostic impacts of CS-CTCs across various subgroups (Additional File 1, Supplementary Fig. 5 and Supplementary Tables 9–11), albeit at low detection rates. Intriguingly, even with the low detection frequency, the stage-dependent effects of CS-CTCs were congruent with the observations made in DLA CS-CTCs (Fig. 2E **and H**). Taken together, the prognostic data suggest that CS-CTCs could serve as a useful biomarker to select M0-patients for intensified multimodal treatments and to guide cancer-directed surgery in the challenging situation of oligometastatic PDAC [20]. For CTC + M1-patients, tumor resection may be contraindicated, whereas for M1-patients negative for DLA-CS-CTCs resection may be beneficial if technically feasible. Nonetheless, this premise requires further validation through prospective clinical studies.

## Conclusion

Our findings emphasize the clinical significance of CS-CTCs in PDAC, highlighting the necessity of high-volume blood analysis for enhanced detection. This approach complements and potentially surpasses other circulating biomarkers in PDAC, such as ctDNA. Even with the limitation of our study restricting analysis to 5% of DLA products, our CS-CTC detection align with ctDNA detection rates even in advanced PDAC [21, 22]. This can be attributed to minimal cfDNA shedding in PDAC and the challenges associated with obtaining tissue in non-operable cases for tumor-informed approaches. While ctDNA analysis is immensely valuable, it predominantly leans towards quantification in PDAC due to these limitations. Conversely, DLA CTCs open avenues for versatile qualitative analyses, addressing clinically relevant diagnostic tests with the potential even enabling functional CTC studies [23]. Emerging technical solutions for analyzing entire DLA products will further amplify sensitivity [10, 17]. Leukapheresis, despite its complexity compared to conventional blood draws, is clinically routine and, as exploited in our study, provides a practical method to obtain high-quality CTC-material. Implementing this ultra-sensitive approach in newly diagnosed PDAC patients could enable CTC-based therapeutic decision-making, facilitating nuanced staging and risk stratification for personalized surgical treatment. Our findings pave the way for subsequent prospective clinical trials, exploring the DLA approach either independently or synergistically with other circulating biomarkers to refine liquid biopsies for PDAC patients.

### Electronic supplementary material

Below is the link to the electronic supplementary material.

Supplementary Material 1. Description of data: This file contains Supplementary Methods, Supplementary Tables 1-11 and Supplementary Figures 1-5 as supporting information and data for the main manuscript.

## Data Availability

The datasets used and/or analyzed during the current study are available from the corresponding author on reasonable request.

## Abbreviations

AUC: area under the curve
CNAs: copy number alterations
CDF: cumulative distribution function
CS: CellSearch
CTCs: circulating tumor cells
CS-CTCs: CS-detected EpCAM+/CK+/CD45−/DAPI + CTCs
DU: Düsseldorf
DLA: diagnostic leukapheresis
EpCAM: epithelial cell adhesion molecule
HE: Heidelberg
HR: hazard ratio
KRAS: Kirsten rat sarcoma viral oncogene homolog
log-rank test: non-parametric statistical test for survival data
M0: absence of distant metastasis
M1: presence of distant metastasis
MNC: Mononuclear cell
OS: overall survival
PB: peripheral blood
PDAC: pancreatic ductal adenocarcinoma
ROC: receiver operating characteristic
SNVs: single nucleotide variants
TP53: tumor protein p53
