# Supplementary material for: Ultra-sensitive CTC-based liquid biopsy for pancreatic cancer enabled by large blood volume analysis
Source: Mol Cancer. 2023 Nov 13;22:181. doi: 10.1186/s12943-023-01880-1 (PMC10641981; doi:10.1186/s12943-023-01880-1)
Supplement: Supplementary file 1 — Supplementary Material 1. Description of data: This file contains Supplementary Methods, Supplementary Tables 1-11 and Supplementary Figures 1-5 as supporting information and data for the main manuscript. [file 12943_2023_1880_MOESM1_ESM.docx]

**Additional File 1, Supplementary Information**

Ultra-sensitive CTC-based Liquid Biopsy for Pancreatic Cancer enabled by Large Blood Volume Analysis

Nikolas H. Stoecklein^1^*, Georg Fluegen^1^*, Rosa Guglielmi^1^*, Rui Neves^1^, Thilo Hackert^2,3^, Emrullah Birgin^4^, Stefan A. Cieslik^1^, Monica Sudarsanam^1^, Christiane Driemel^1^, Guus van Dalum^1^, André Franken^5^, Dieter Niederacher^5^, Hans Neubauer^5^, Tanja Fehm^5^, Jutta M. Rox^6^, Petra Böhme^7^, Lena Häberle^8^, Wolfgang Göring^8^, Irene Esposito^8^, Stefan A. Topp^1^, Frank A.W. Coumans^9^, Jürgen Weitz^10^, Wolfram T. Knoefel^1^, Johannes C. Fischer^6^*, Ulrich Bork^10^*, Nuh N. Rahbari^4,11^*

^1^General, Visceral and Pediatric Surgery University Hospital and Medical Faculty of the Heinrich-Heine University Düsseldorf, Moorenstr. 5, 40225, Düsseldorf, Germany

^2^Department of General, Visceral, and Transplantation Surgery, Heidelberg University Hospital, Im Neuenheimer Feld 420, 69120 Heidelberg, Germany

^3^Current Affiliation: Department of General, Visceral and Thoracic Surgery, University Medical Center Hamburg-Eppendorf, Hamburg, Germany

^4^Department of Surgery, University Hospital Mannheim, Medical Faculty Mannheim, Theodor-Kutzer-Ufer 1-3, 68167 Mannheim, Germany

^5^Department of Obstetrics and Gynecology, University Hospital and Medical Faculty of the Heinrich-Heine University Düsseldorf, Moorenstr. 5, 40225, Düsseldorf, Germany

^6^Department of Transplantation Diagnostics and Cell Therapeutics, University Hospital and Medical Faculty of the Heinrich-Heine University Düsseldorf, Moorenstr. 5, 40225, Düsseldorf, Germany

^7^Institute of Forensic Medicine Düsseldorf, University Hospital and Medical Faculty of the Heinrich-Heine University Düsseldorf, Moorenstr. 5, 40225, Düsseldorf, Germany

^8^Institute of Pathology, University Hospital and Medical Faculty of the Heinrich-Heine University Düsseldorf, Moorenstr. 5, 40225, Düsseldorf, Germany

^9^Decisive Science, Ertskade10, 1019BB Amsterdam, The Netherlands

^10^Department of Visceral, Thoracic and Vascular Surgery, University Hospital Carl Gustav Carus of the Technical University Dresden, Fetscherstr. 74, 01307 Dresden, Germany

^11^Current Affiliation: Department for General and Visceral Surgery, University Hospital Ulm, Albert-​Einstein-Allee 23, 89081 Ulm, Germany

*These authors contributed equally

**Supplementary Methods**

*Patient cohorts*

Patients over 18 years with histologically proven PDAC who were scheduled for surgical exploration with the aim of potentially curative resection were eligible for inclusion in the study. Patients were excluded if they received treatment for another malignancy within the past 5 years. For patients screened for DLA, further exclusion criteria were an insufficient bilateral cubital vein status to allow puncture, coagulation disorders or any other condition not allowing leukapheresis. From a first cohort of 170 patients treated at the Department of General, Visceral and Transplantation Surgery, Heidelberg University Hospital, Germany, between 2009 and 2012 we collected only PB samples (HE-cohort, **Figure 1A**). In a second cohort of 60 PDAC patients (DU-cohort, **Figure 1A**), admitted and treated between 2011 and 2023 at the Department of General, Visceral and Pediatric Surgery, University Hospital of the Heinrich-Heine-University Duesseldorf, Germany, we performed DLA (DU-DLA-cohort) and collected matched peripheral blood (DU-PB-cohort) prior DLA. Patients in the D-cohort had a higher proportion of pT1/2 tumors (p<0.001), R0-resections (p<0.001) and distant metastases (M1, p=0.004) while the remaining clinicopathological characteristics were comparable between both cohorts. Clinical follow-up data for overall survival (OS) were limited to 60 months. The studies were independently approved by the Ethics Committees of the Medical Faculty of the Heinrich-Heine University Duesseldorf and the Ruprecht-Karls-University Heidelberg, respectively. All patients included into the study provided written informed consent.

*Diagnostic Leukapheresis (DLA)*

We performed DLA in 60 PDAC patients at the Department of Transplantation Diagnostics and Cell Therapeutics, Duesseldorf, Germany, as previously described [1, 2]. For all 60 patients with completed DLA (**Figure 1A**) WBC counts were obtained for PB and the DLA products. The equivalent PB volume processed in each DLA CS assay was calculated by dividing the product of the MNC concentration in DLA and the volume of the DLA aliquot used by the concentration of MNCs measured in the same patient's PB sample. For two patients PB samples could not be analyzed and are missing (58 datapoints for PB CS-CTC analyses in the DU-cohort).

*CTC detection in peripheral blood and DLA-products using CellSearch (CS)*

Detecting CTCs with CS was performed according to the manufacturer's guidelines provided by Menarini Silicon Biosystems (Bologna, Italy), and was adapted for use with DLA samples as previously described [1, 2]. The fluorescence images from DLA samples were analyzed using the ACCEPT software v1.1 and the "Full Detection" function, since this provides a better segmentation algorithm compared to the original CS System [3], which can be advantageous in more dense CS-cartridges from DLA samples [3]. The linear relaxed gates used to identify potential CTCs in the images are outlined in **Supplementary Table 1**. The gated objects presented as thumbnail image galleries together with the extracted cellular contour and measurements were reviewed independently by two experienced operators to determine the CTCs. ACCEPT was not used for fully automated CTC detection.

*Genetic characterization of single CTCs*

Cryopreserved DLA material was thawed, processed, and analyzed with CS as previously described [4]. ACCEPT was applied to CS images as described above to identify CTCs. Isolation of individual cells (CTCs and WBCs) by FACS from CS cartridges was followed by single cell whole genome amplification (WGA) and quality control of generated WGA products exactly as described by Neves et al. [5]. Only WGA products with ≥3 bands in the quality control polymerase chain reaction (PCR) were subjected for further analysis. For amplicon based NGS of the WGA products we followed the protocol for PCRs and data analysis provided by Franken et al. [6]. The primers for KRAS and TP53 are listed in **Supplementary Table 2.** To obtain copy number alteration (CNA) profiles from the WGA products, we used the Ampli1 LowPass Kit for Illumina platforms (Menarini Silicon Biosystems, Bologna, Italy). Library amplification, indexing, quality controls, and single-read sequencing on the MiSeq (illumina, San Diego, USA, Ca) were performed according to the manufacturer’s instructions.

*Cumulative distribution functions*

For extrapolating the fraction of samples that have at least 1 or N CTC when analyzing a larger fraction of the collected DLA product, cumulative distribution functions (CDF) were fit to the empirical cumulative distribution functions in python 3.8 with statsmodels 0.13.5. The CTC data were derived from the DU-PB and matched DU-DLA cohorts. The log-logistic function was fit to whole blood and DLA samples CDFs via maximum likelihood estimation to the empirical curve as preciously described by Coumans et al [7].

*Survival analysis*

Survival analysis was performed using the 'survival' package (version 3.5-5) in R (version 4.3.0), with all relevant parameters calculated, such as hazard ratio and log-rank p-value. To evaluate multiple cutoff values, a loop was utilized to compute these parameters using a vector file containing unique values. The optimal cutoff value was determined by employing a sorting function. Additionally, area under the curve (AUC) values were computed using the 'pROC' package (version 1.18.2) in R, which was also implemented within a loop to enhance the analysis.

*Bioinformatic analysis of NGS data*

Whole genome sequences (WGS) from CTCs or FFPE samples were aligned to the human reference genome (hg19) using Burrows-Wheeler Alignment Tool (BWA 0.7.15). CNAs were predicted by using QDNAseq 11.0 with a window size of 500 kb. “Gain” and “loss” calls were filtered out by residual (> 4 standard deviations, SD i.e a default setting) and segmented copy number data of each sample were extracted in log2Ratio values. To assess the quality of the generated profiles, sequence-aligned reads were randomly subsampled closer to 200,000 reads and copy number analysis was performed with QDNAseq using a window size of 500 kb. Derivative Log Ratio Spread (DLRS) was then calculated since high DLRS values suggest a high level of noise (low-quality). Samples with DLRS values ≥ 0.35 were labeled as “failed” as the detected copy-numbers were not reliable. Similarly, Interquartile range (IQR) values were calculated, and samples were marked as failed if the IQR of the log-ratios was ≥ 0.35. Samples were classified as aberrant if the whole genome alteration percentage (GAP) was above 2.5%. To validate the threshold of GAP, we calculated the whole-genome altered percentage for a dataset of WBCs, and the average of the GAP, i.e., 2.5% was considered as a threshold to determine a sample as an aberrant profile.

**Supplementary Table 1**: Channels and gates used in ACCEPT to identify potential CTCs for review by trained operators.

|  | **DLA ("Candidate dla")** |
| --- | --- |
| Channel | Gate |
| CD45 (APC) Mean Intensity | 20 |
| Nucleus (DAPI) Mean Intensity | 100 |
| CK (PE) Mean Intensity | 20 |
| CK (PE) Size | 500 |
| CK (PE) Size | 20 |
| Overlay with Nucleus | - |

**Supplementary Table 2:** Primers used for amplicon based NGS

| **Gene** | **Exon** | **Amplicon Size** | **Forward Primer** |
| --- | --- | --- | --- |
| KRAS | 2 | 208 | AAGACTCGGCAGCATCTCCATTGGATCATATTCGTCCACAA |
| KRAS | 3 | 232 | AAGACTCGGCAGCATCTCCACCTTCTCAGGATTCCTACAG |
| TP53 | 5 | 233 | AAGACTCGGCAGCATCTCCACTACAGTACTCCCCTGCCCT |
| TP53 | 7 | 232 | AAGACTCGGCAGCATCTCCAGACTGTACCACCATCCACTA |
| TP53 | 8 | 219 | AAGACTCGGCAGCATCTCCATGCCTCTTGCTTCTCTTTTC |
| **Gene** | **Exon** | **Amplicon Size** | **Reverse Primer** |
| KRAS | 2 | 208 | GCGATCGTCACTGTTCTCCACCTTATGTGTGACATGTTCTAATATAGT |
| KRAS | 3 | 232 | GCGATCGTCACTGTTCTCCACCACCTATAATGGTGAATATC |
| TP53 | 5 | 233 | GCGATCGTCACTGTTCTCCACACCATCGCTATCTGAGCA |
| TP53 | 7 | 232 | GCGATCGTCACTGTTCTCCAAGAAATCGGTAAGAGGTGGG |
| TP53 | 8 | 219 | GCGATCGTCACTGTTCTCCACTTGCTTACCTCGCTTAGTG |

**Supplementary Table 3**

|  | **DU-cohort** | | | **HE-cohort** | |
| --- | --- | --- | --- | --- | --- |
|  |  | **PB** | **DLA** | **PB** | |
|  | **Total**  **n=60** | **CTC+^a^**  **n=11 (19%)** | **CTC+^b^**  **n=32 (53%)** | **Total**  **n=170** | **CTC+^a^**  **n=16 (9%)** |
| **Time of observation**  Median follow-up in months (range) | 12.7  (0.1-131.4) | 6.8  (0.6-43.9) | 7.0  (0.1-67.4) | 15.0  (0.1-84.0) | 9.0  (1.0-40.0) |
| **Median age** in years (range) | 65.5 (40-86) | 58 (46-73) | 68.5 (44-86) | 66 (33-88) | 61.5 (40-85) |
| **Sex**  Male  Female | 30 (50%)  30 (50%) | 6 (55%)  5 (45%) | 16 (50%)  16 (50%) | 72 (42%)  98 (58%) | 6 (38%)  10 (62%) |
| **UICC**  I-III  IV | 41 (68%)  19 (32%) | 4 (36%)  7 (64%) | 18 (56%)  14 (44%) | 146 (86%)  24 (14%) | 12 (75%)  4 (25%) |
| **Grade of differentiation^c^**  G1-2  G3-4 | 35 (65%)  19 (35%) | 5 (50%)  5 (50%) | 18 (60%)  12 (40%) | 71 (59%)  49 (41%) | 5 (46%)  6 (54%) |
| **Surgery with curative intention**  Yes  No | 45 (75%)  15 (25%) | 5 (45%)  6 (55%) | 21 (66%)  11 (34%) | 128 (75%)  42 (25%) | 12 (75%)  4 (25%) |
| **TNM-Classification^c,d^**  pT1-2  pT3-4 | 19 35%)  36 (65%) | 5 (50%)  5 (50%) | 9 (32%)  19 (68%) | 7 (6%)  120 (94%) | 0 (0%)  12 (100%) |
| pN0  pN1-2 | 8 (15%)  46 (85%) | 2 (22%)  7 (78%) | 4 (14%)  24 (86%) | 22 (17%)  105 (83%) | 2 (17%)  10 (83%) |
| **Operation with R0-resection^c^**  Yes  No | 38 (69%)  17 (31%) | 3 (30%)  7 (70%) | 16 (55%)  13 (45%) | 26 (21%)  100 (79%) | 3 (25%)  9 (75%) |

**Supplementary Figure 1**: DLA findings: Equivalent PB volumes calculated from the input volume and the MNC concentration of the DLA products analyzed in CS. The dotted line indicates the median PB equivalent and the filled circles CTC-positivity.

**
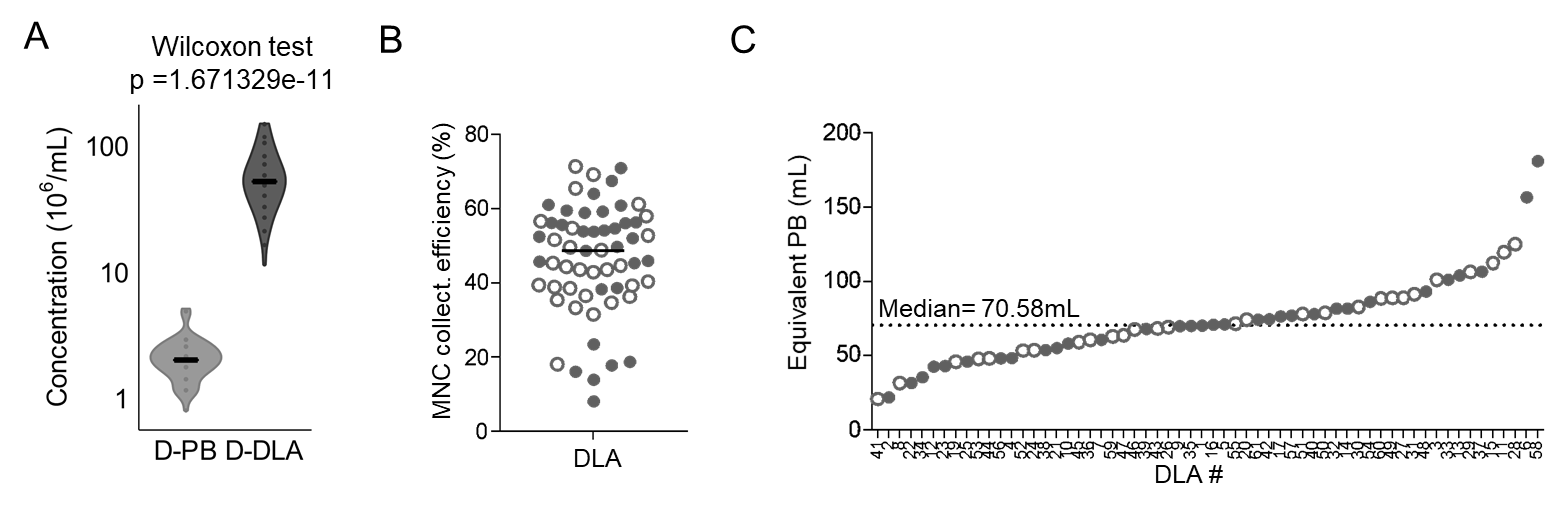
**

**Supplementary Figure 2:** CS re-analysis of thawed DLA samples (n=8) in comparison the initial CS-CTC count in fresh DLA material (Wilcoxon test: p>0.5). Filled circles represent samples with successfully analyzed CNA-positive CTCs.


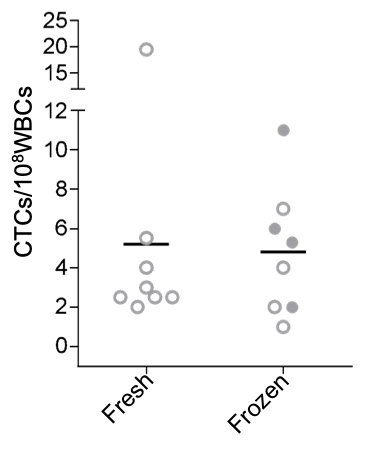


**Supplementary Table 4**: low-pass profiling and amplicon-based panel NGS (single CTCs, single WBC, micro-dissected bulk tumor tissue)

| **Patient #** | **Sample** | **CNV profile** | **TP53/KRAS Mutational Status** | | |
| --- | --- | --- | --- | --- | --- |
|  |  |  | **Mutation** | **VAF** | **Classification** |
| **patient 2** | CTC | aberrant | WT |  | - |
|  | WBC | ND | WT |  | - |
| **patient 7** | CTC | aberrant | G12V (KRAS) | 100 | pathogenic |
|  | CTC | aberrant | G12V (KRAS) | 100 | pathogenic |
|  | CTC | aberrant | G12V (KRAS) | 100 | pathogenic |
|  | CTC | aberrant | G12V (KRAS) | 16 | pathogenic |
|  | CTC | aberrant | G12V (KRAS) | 100 | pathogenic |
|  | CTC | aberrant | G12V (KRAS) | 100 | pathogenic |
|  | WBC | ND | WT |  |  |
|  | WBC | ND | WT |  |  |
|  | WBC | ND | WT |  |  |
|  | WBC | ND | WT |  |  |
|  | Met (PUL) | NA | G12V (KRAS); G12D (KRAS); D186N (TP53); R273H (TP53); E285K (TP53) | 1.76; 0.48; 1.12; 1.38; 1.25 | All pathogenic |
| **patient 17** | CTC | aberrant | G12V (KRAS) | 50 | pathogenic |
|  | CTC | aberrant | G12V (KRAS) | 100 | pathogenic |
|  | CTC | aberrant | P112R (TP53) | 31 | pathogenic |
|  | WBC | ND | WT |  |  |
|  | WBC | not aberrant | WT |  |  |
|  | WBC | ND | WT |  |  |
|  | WBC | not aberrant | NA |  |  |
|  | Met (HEP) | aberrant | G12R (KRAS); P151R (TP53) | 21;25 | All pathogenic |
| **patient 22** | CTC | aberrant | WT |  |  |
|  | WBC | not aberrant | WT |  |  |
|  | WBC | ND | WT |  |  |
|  | WBC | ND | WT |  |  |
|  | Tumor  (PT) | not aberrant | WT |  |  |

ND: not done; NA: not available; Met PUL: pulmonary metastasis; Met HEP: hepatic metastasis; PT: primary PDAC tumor; WT: wild type; 14 unmatched WBC: WT in TP53/KRAS.

**Supplementary Table 5:** Prognostic impact on OS of CS-CTCs and of clinico-pathological parameters in the DU-DLA cohort (n=60)

|  | ***univariate Analysis*** | | | ***multivariable Analysis*** | | |
| --- | --- | --- | --- | --- | --- | --- |
| **Risk factor** | **Relative**  **risk** | **95% Confidence interval** | ***P*-value** | **Hazard ratio** | **95% Confidence interval** | ***P*-value** |
| Age (median; ≤66 years vs. >66 years) | 1.13 | 0.62-2.04 | 0.697 | - | - | - |
| Gender (male vs. female) | 0.91 | 0.50-1.65 | 0.751 | - | - | - |
| UICC III-IV vs. I-II | 2.71 | 1.44-5.12 | **0.002** | - | - | NS |
| G1/2 vs. G3/4 | 0.94 | 0.49-1.79 | 0.850 | - | - | - |
| R1/2 vs. R0 | 2.30 | 1.24-4.26 | **0.008** | - | - | NS |
| PB CTC+ vs. PB CTC-  *≥1CTC/7.5mL* | 1.45 | 0.67-3.15 | 0.349 | - | - | - |
| PB CTC+ vs. PB CTC-  *≥3CTC/7.5mL* | 4.95 | 1.70-14.46 | **0.003** | - | - | NS* |
| DLA CTC+ vs. DLA CTC-  *≥1CTC/~70mL* | 2.71 | 1.45-5.04 | **0.002** | 2.14 | 1.06-4.33 | **0.034*** |
| DLA CTC+ vs. DLA CTC-  *≥3CTC/~70mL* | 1.91 | 1.05-3.46 | **0.033** | - | - | NS* |

*PB and DLA as well as ≥3CTC and ≥1CTC were tested separately upon multivariable analysis

**Supplementary Figure 3:** Impact on OS of CS-CTCs detected in DLA-products of PDAC patients for the subgroup treated with curative intention (A and B, n=45) and for the M0 group (C and D, n=41). CS-CTC positive versus CS-CTC negative, A and C: red line: ≥1CTCs/~70mL of blood, green line: 0CTCs/~70mL of blood; B and D: red line: ≥3CTCs/~70mL of blood, green line: <3CTCs/~70mL of blood.


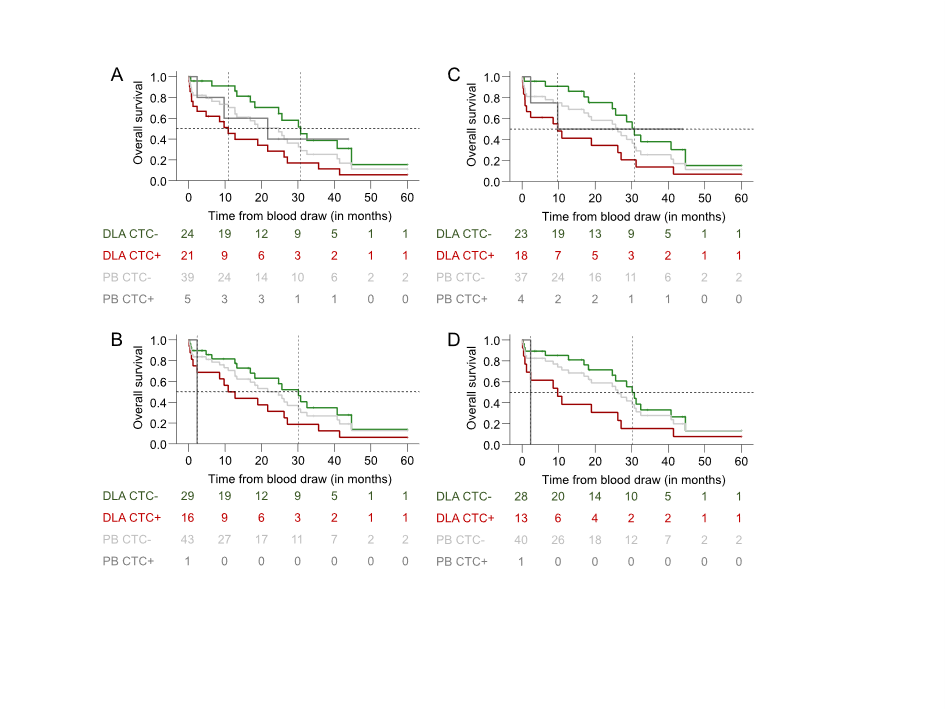


**Supplementary Table 6:** Prognostic impact on OS of CS-CTCs and of clinico-pathological parameters in the DU-DLA cohort treated with curative intention (n=45)

|  | ***univariate Analysis*** | | | ***multivariable Analysis*** | | |
| --- | --- | --- | --- | --- | --- | --- |
| **Risk factor** | **Relative**  **risk** | **95% Confidence interval** | ***P*-value** | **Hazard ratio** | **95% Confidence interval** | ***P*-value** |
| Age (median; ≤66 years vs. >66 years) | 1.08 | 0.53-2.20 | 0.825 | - | - | - |
| Gender (male vs. female) | 0.82 | 0.40-1.67 | 0.584 | - | - | - |
| UICC III-IV vs. I-II | 2.20 | 0.98-4.93 | 0.055 | - | - | - |
| G1/2 vs. G3/4 | 0.98 | 0.45-2.09 | 0.949 | - | - | - |
| R1/2 vs. R0 | 1.70 | 0.69-4.17 | 0.248 | - | - | - |
| PB CTC+ vs. PB CTC-  *≥1CTC/7.5mL* | 0.82 | 0.25-2.70 | 0.742 | - | - | - |
| PB CTC+ vs. PB CTC-  *≥3CTC/7.5mL* | 5.605 | 0.689-45.58 | 0.106 | - | - | - |
| DLA CTC+ vs. DLA CTC-  *≥1CTC/~70mL* | 2.55 | 1.24-5.24 | **0.011** | - | - | -* |
| DLA CTC+ vs. DLA CTC-  *≥3CTC/~70mL* | 1.91 | 1.05-3.46 | **0.033** | - | - | -* |

*PB and DLA as well as ≥3CTC and ≥1CTC were tested separately upon multivariable analysis

**Supplementary Table 7:** Prognostic impact on OS of CS-CTCs and of clinico-pathological parameters in the DU-DLA M0 subgroup (n=41)

|  | ***univariate Analysis*** | | |
| --- | --- | --- | --- |
| **Risk factor** | **Relative**  **risk** | **95% Confidence interval** | ***P*-value** |
| Age (median; ≤66 years vs. >66 years) | 1.26 | 0.59-2.68 | 0.546 |
| Gender (male vs. female) | 0.69 | 0.32-1.49 | 0.348 |
| G1/2 vs. G3/4 | 0.976 | 0.44-2.18 | 0.952 |
| R1/2 vs. R0 | 1.418 | 0.57-3.55 | 0.455 |
| PB CTC+ vs. PB CTC-  *≥1CTC/7.5ml* | 0.718 | 0.17-3.04 | 0.718 |
| PB CTC+ vs. PB CTC-  *≥3CTC/7.5ml* | 5.196 | 0.64-42.25 | 0.123 |
| DLA CTC+ vs. DLA CTC-  *≥1CTC/~70mL* | 2.48 | 1.17-5.25 | **0.017** |
| DLA CTC+ vs. DLA CTC-  *≥3CTC/~70mL* | 2.219 | 1.04-4.72 | **0.038** |

**Supplementary Table 8**: Evaluation metrics for CS-CTCs to predict overall survival (death) in DLA and PB samples of PDAC patients and of the subgroup undergoing treatment with curative intention. For this analysis we consider True Positives as patients with CTCs who died within the observation period. False Positives: Patients with CTCs who did not die within the observation period. True Negatives: Patients without CTCs who did not die within the observation period. False Negatives: Patients without CTCs who died within the observation period.

|  | **DU-DLA**  **n=60**  **(~70mL blood)** | | **DU-DLA curative intention**  **n=45**  **(~70mL blood)** | | **DU-PB**  **n=58**  **(7.5mL blood)** | | **DU-PB**  **curative intention**  **n=44**  **(7.5mL blood)** | |
| --- | --- | --- | --- | --- | --- | --- | --- | --- |
|  | *CTC≥1* | *CTC≥3* | *CTC≥1* | *CTC≥3* | *CTC≥1* | *CTC≥3* | *CTC≥1* | *CTC≥3* |
| **Sensitivity** | 64% | 48% | 58% | 48% | 19% | 12% | 10% | 3% |
| **Specificity** | 75% | 88% | 79% | 93% | 81% | 94% | 86% | 100% |
| **Accuracy** | 67% | 58% | 64% | 62% | 36% | 34% | 34% | 34% |
| **Prevalence** | 73% | 73% | 69% | 69% | 72% | 72% | 68% | 68% |
| **Positive Predictive Value (PPV)** | 88% | 91% | 86% | 94% | 73% | 83% | 60% | 100% |
| **Negative Predictive Value (NPV)** | 43% | 38% | 46% | 45% | 28% | 29% | 31% | 33% |
| **Post-Test Disease Probability** | 81% | 80% | 78% | 79% | 73% | 75% | 65% | 76% |
| **Post-Test Health Probability** | 62% | 72% | 63% | 80% | 50% | 62% | 45% | 100% |
| **Positive Likelihood Ratio** | 2.55 | 3.82 | 2.71 | 6.77 | 1.02 | 1.90 | 0.7 | / |
| **Negative Likelihood Ratio** | 0.48 | 0.60 | 0.53 | 0.56 | 1.00 | 0.94 | 1.05 | 0.97 |

**Supplementary Figure 4**: ROC curves and corresponding AUC values for the top 10 DLA CS-CTC cutoffs, separately for M0 and M1 patients of DLA cohort. These cutoffs were selected based on their combined highest hazard ratio and AUC value of M0 (**A**, n = 41) and M1 (**B**, n = 19) PDAC patients. Based on the criteria, the cutoff at ≥3CTC/~70mL (p=0.034; HR: 2.22) was selected for M0-patients and the only significant cutoff for M1-patients at ≥23CTC/~70mL (p=0.04; HR: 4.79). The cutoffs were used in the analysis of Figure 2E.


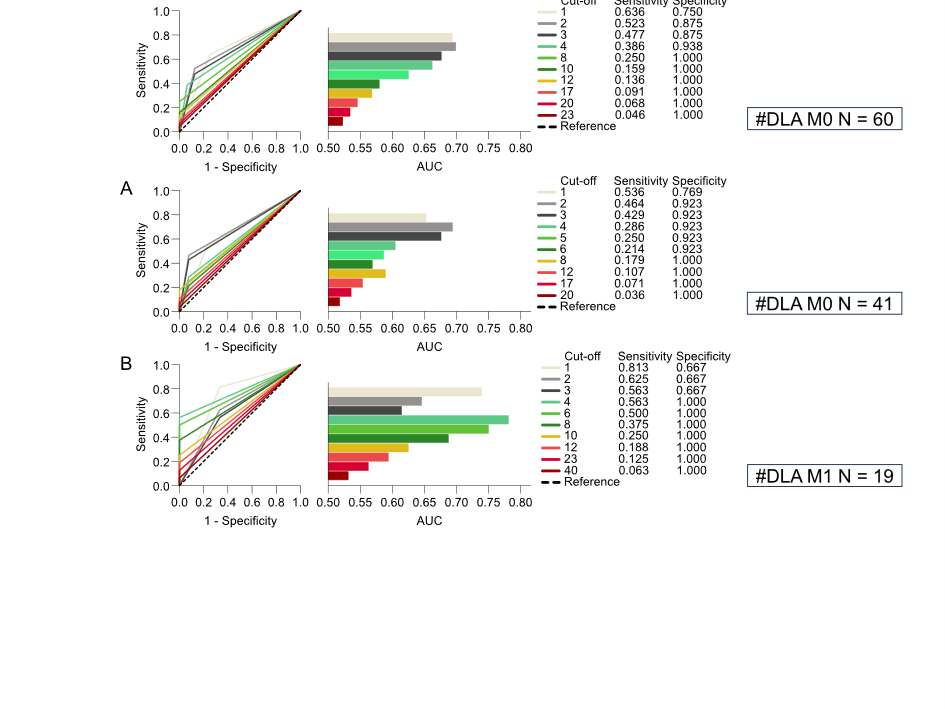


**Supplementary Table 9**: Prognostic impact on OS of clinico-pathological parameters in the combined HE/DU-cohort (n=228).

|  | ***univariate Analysis*** | | | ***multivariable Analysis*** | | |
| --- | --- | --- | --- | --- | --- | --- |
| **Risk factor** | **Relative**  **risk** | **95% Confidence interval** | ***P*-value** | **Hazard ratio** | **95% Confidence interval** | ***P*-value** |
| Age (median; ≤66 years vs. >66 years) | 0.98 | 0.72-1.33 | 0.873 | - | - | - |
| Gender (male vs. female) | 0.88 | 0.64-1.19 | 0.401 | - | - | - |
| UICC III-IV vs. I-II | 2.35 | 1.68-3.28 | **<0.001** | 2.39 | 1.58-3.63 | **<0.001** |
| G1/2 vs. G3/4 | 1.40 | 0.99-1.99 | 0.056 | - | - | - |
| R1/2 vs. R0 | 1.52 | 1.12-2.06 | **0.007** | - | - | NS |
| PB CTC+ vs. PB CTC-  *≥1CTC/7.5ml* | 1.94 | 1.25-3.03 | **0.003** | 1.864 | 1.18-2.95 | **0.008** |
| PB CTC+ vs. PB CTC-  *≥3CTC/7.5ml* | 7.72 | 3.58-16.63 | **<0.001** | 5.20 | 2.23-12.12 | **<0.001*** |

*Multivariable analysis for ≥3CTC/7.5mL and for ≥1CTC/7.5mL were performed separately

**Supplementary Table 10**: Prognostic impact on OS of clinico-pathological parameters in the HE-cohort (n=170).

|  | ***univariate Analysis*** | | | ***multivariable Analysis*** | | |
| --- | --- | --- | --- | --- | --- | --- |
| **Risk factor** | **Relative**  **risk** | **95% Confidence interval** | ***P*-value** | **Hazard ratio** | **95% Confidence interval** | ***P*-value** |
| Age (median; ≤66 years vs. >66 years) | 0.92 | 0.65-1.32 | 0.662 | - | - | - |
| Gender (male vs. female) | 0.86 | 0.60-1.24 | 0.421 | - | - | - |
| UICC III-IV vs. I-II | 2.11 | 1.42-3.15 | **<0.001** | 3.16 | 1.54-6.47 | **0.002/**  **0.001*** |
| G1/2 vs. G3/4 | 1.65 | 1.01-2.51 | **0.020** | 1.60 | 1.04-2.45 | **0.032/**  **0.025*** |
| R1/2 vs. R0 | 2.16 | 1.20-3.88 | **0.010** | - | - | NS/  **0.047*** |
| PB CTC+ vs. PB CTC-  *≥1CTC/7.5ml* | 2.21 | 1.28-3.82 | **0.003** | - | - | NS |
| PB CTC+ vs. PB CTC-  *≥3CTC/7.5ml* | 11.63 | 3.50-38.64 | **<0.001** | 10.32 | 1.26-84.48 | **0.030*** |

*Multivariable analysis for ≥3CTC/7.5mL and for ≥1CTC/7.5mL were performed separately

**Supplementary Table 11:** Prognostic impact of clinico-pathological parameters on OS in the HE-cohort treated with curative intend (n=128).

|  | ***univariate Analysis*** | | | ***multivariable Analysis*** | | |
| --- | --- | --- | --- | --- | --- | --- |
| **Risk factor** | **Relative**  **risk** | **95% Confidence interval** | ***P*-value** | **Hazard ratio** | **95% Confidence interval** | ***P*-value** |
| Age (median; ≤66 years vs. >66 years) | 0.90 | 0.58-1.33 | 0.54 | - | - | - |
| Gender (male vs. female) | 0.88 | 0.57-1.30 | 0.48 | - | - | - |
| UICC III-IV vs. I-II | 2.92 | 1.49-5.49 | **0.002** | 3.13 | 1.52-6.42 | **0.002/**  **0.001*** |
| G1/2 vs. G3/4 | 1.65 | 1.08-2.51 | **0.021** | 1.60 | 1.04-2.46 | **0.034/**  **0.026*** |
| R1/2 vs. R0 | 2.06 | 1.15-3.74 | **0.015** | - | - | NS/  **0.048*** |
| PB CTC+ vs. PB CTC-  *≥1CTC/7.5ml* | 2.14 | 1.16-3.95 | **0.012** | - | - | NS |
| PB CTC+ vs. PB CTC-  *≥3CTC/7.5ml* | 8.79 | 2.04-37.80 | **<0.001** | 10.17 | 1.24- 83.27 | **0.031*** |

*Multivariable analysis for ≥3CTC/7.5mL and for ≥1CTC/7.5mL were performed separately

**Supplementary Figure 5**: Impact on OS of CS-CTC detected in peripheral blood samples for the subgroups treated with curative intention (p-values from log-rank tests). A-C: cutoff for CTC-positivity: ≥1CTC/7.5mL, D-F: cutoff for CTC-positivity: ≥3CTC/7.5mL; A and D, HE-cohort (n=128); B and E, pooled HE- and DU-cohorts (n=172), C and F, pooled HE- and DU-cohort (n=172) separated by M-status.


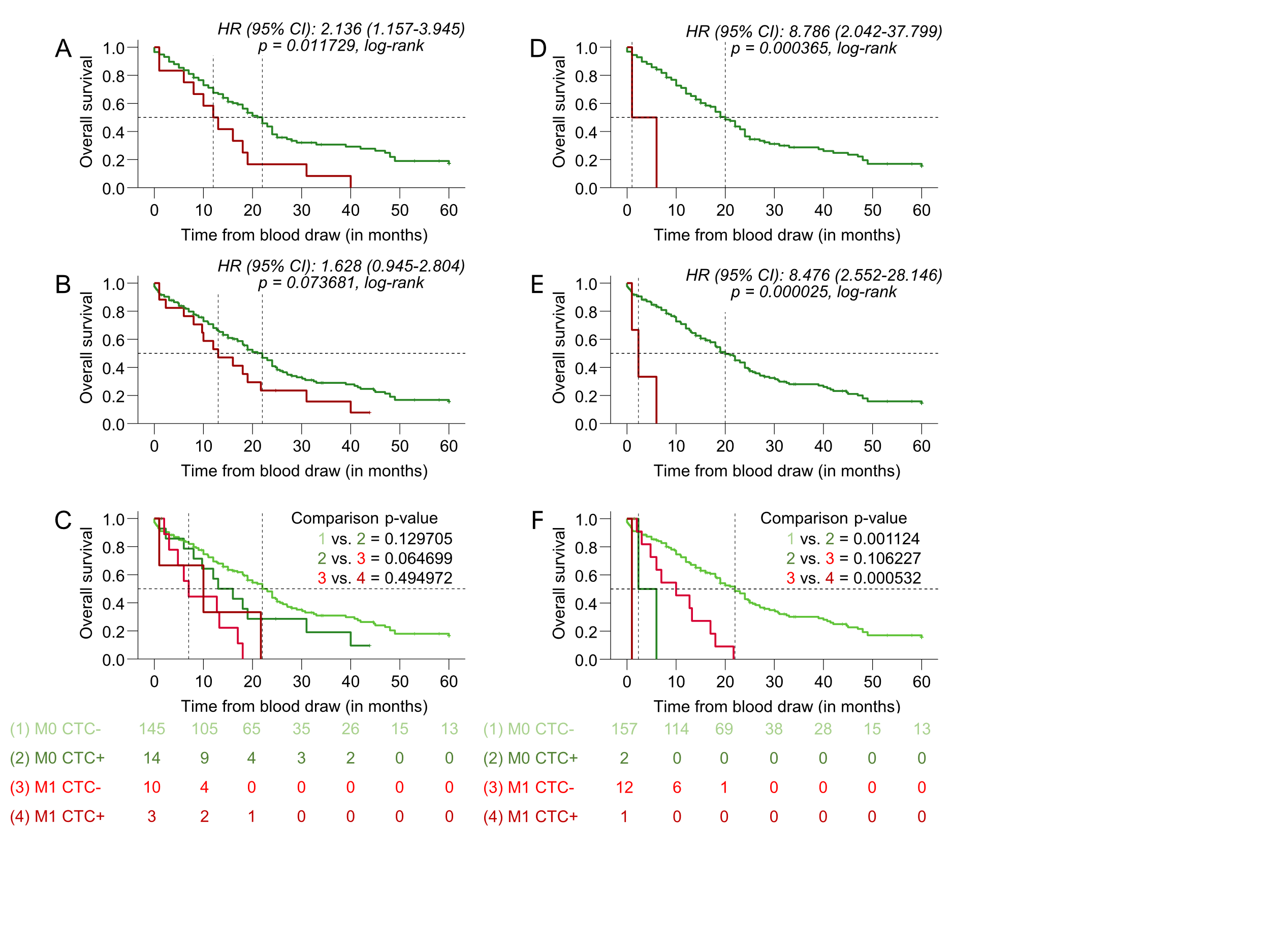


**References**

1. Fehm TN, Meier-Stiegen F, Driemel C, Jager B, Reinhardt F, Naskou J, Franken A, Neubauer H, Neves RPL, van Dalum G, et al: **Diagnostic leukapheresis for CTC analysis in breast cancer patients: CTC frequency, clinical experiences and recommendations for standardized reporting.** *Cytometry A* 2018, **93:**1213-1219.

2. Fischer JC, Niederacher D, Topp SA, Honisch E, Schumacher S, Schmitz N, Zacarias Fohrding L, Vay C, Hoffmann I, Kasprowicz NS, et al: **Diagnostic leukapheresis enables reliable detection of circulating tumor cells of nonmetastatic cancer patients.** *Proc Natl Acad Sci U S A* 2013, **110:**16580-16585.

3. Stevens M, Mentink A, Nanou A, Coumans FAW, Isebia KT, Kraan J, Hamberg P, Martens JWM, Terstappen L: **Improved enrichment of circulating tumor cells from diagnostic leukapheresis product.** *Cytometry A* 2023.

4. Franken A, Driemel C, Behrens B, Meier-Stiegen F, Endris V, Stenzinger A, Niederacher D, Fischer JC, Stoecklein NH, Ruckhaeberle E, et al: **Label-Free Enrichment and Molecular Characterization of Viable Circulating Tumor Cells from Diagnostic Leukapheresis Products.** *Clin Chem* 2019, **65:**549-558.

5. Neves RP, Raba K, Schmidt O, Honisch E, Meier-Stiegen F, Behrens B, Mohlendick B, Fehm T, Neubauer H, Klein CA, et al: **Genomic high-resolution profiling of single CKpos/CD45neg flow-sorting purified circulating tumor cells from patients with metastatic breast cancer.** *Clin Chem* 2014, **60:**1290-1297.

6. Franken A, Honisch E, Reinhardt F, Meier-Stiegen F, Yang L, Jaschinski S, Esposito I, Alberter B, Polzer B, Huebner H, et al: **Detection of ESR1 Mutations in Single Circulating Tumor Cells on Estrogen Deprivation Therapy but Not in Primary Tumors from Metastatic Luminal Breast Cancer Patients.** *J Mol Diagn* 2020, **22:**111-121.

7. Coumans FA, Ligthart ST, Uhr JW, Terstappen LW: **Challenges in the enumeration and phenotyping of CTC.** *Clin Cancer Res* 2012, **18:**5711-5718.
